# Technology-Based Interventions for Substance Use Treatment Among People Who Identify as African American or Black, Hispanic or Latino, and American Indian or Alaska Native: Scoping Review

**DOI:** 10.2196/53685

**Published:** 2024-12-03

**Authors:** Sarah K Moore, Jesse S Boggis, Phoebe R Gauthier, Chantal A Lambert-Harris, Emily G Hichborn, Kathleen D Bell, Elizabeth C Saunders, LaTrice Montgomery, Eilis I Murphy, Avery M Turner, Nico Agosti, Bethany M McLeman, Lisa A Marsch

**Affiliations:** 1 Center for Technology and Behavioral Health, Geisel School of Medicine, Dartmouth College Lebanon, NH United States; 2 The Dartmouth Institute for Health Policy and Clinical Practice, Geisel School of Medicine, Dartmouth College Lebanon, NH United States; 3 College of Medicine, University of Cincinnati Cincinatti, OH United States

**Keywords:** technology-based interventions, substance use, treatment, health equity, underrepresented, scoping review, mobile phone

## Abstract

**Background:**

In the United States, racial and ethnic disparities in substance use treatment outcomes are persistent, especially among underrepresented minority (URM) populations. Technology-based interventions (TBIs) for substance use treatment show promise in reducing barriers to evidence-based treatment, yet no studies have described how TBIs may impact racial or ethnic health equity.

**Objective:**

This study explored whether TBIs in substance use treatment research promote health equity among people who identify as African American or Black, Hispanic or Latino, and American Indian or Alaska Native through their inclusion in research. We explored whether research that includes the aforementioned groups consciously considers race and/or ethnicity beyond including these populations as participants.

**Methods:**

We conducted a scoping review of 5 electronic databases to identify TBIs in substance use treatment studies published in English between January 2000 and March 2021. Studies were included if ≥50% of participants identified as African American or Black, Hispanic or Latino, or American Indian or Alaska Native *when combined*. Included studies were evaluated for conscious consideration of race and ethnicity in at least one manuscript section. Finally, we conducted a critical appraisal of each study’s potential to facilitate insights into the impact of a TBI for members of specific URM groups.

**Results:**

Of 6897 titles and abstracts screened and 1158 full-text articles assessed for eligibility, nearly half (532/1158, 45.9%) of the full-text articles were excluded due to the absence of data on race, ethnicity, or not meeting the aforementioned demographic eligibility criteria. Overall, 110 studies met the inclusion criteria. Study designs included 39.1% (43/110) randomized trials, and 35.5% (39/110) feasibility studies. In total, 47.3% (52/110) of studies used computer-based interventions, including electronic screening, brief interventions, and referrals to treatment, whereas 33.6% (37/110) used interactive voice response, ecological momentary assessment or interventions, or SMS text messaging via mobile phones. Studies focused on the following substances: alcohol or drugs (45/110, 40.9%), alcohol alone (26/110, 23.6%), opioids (8/110, 7.3%), cannabis (6/110, 5.5%), cocaine (4/110, 3.6%), and methamphetamines (3/110, 2.7%). Of the studies that consciously considered race or ethnicity (29/110, 26.4%), 6.4% (7/110) explicitly considered race or ethnicity in all manuscript sections. Overall, 28.2% (31/110) of the studies were critically appraised as having a high confidence in the interpretability of the findings for specific URM groups.

**Conclusions:**

While the prevalence of TBIs in substance use treatment has increased recently, studies that include and consciously consider URM groups are rare, especially for American Indian or Alaska Native and Hispanic or Latino groups. This review highlights the limited research on TBIs in substance use treatment that promotes racial and ethnic health equity and provides context, insights, and direction for researchers working to develop and evaluate digital technology substance use interventions while promoting health equity.

## Introduction

### Background

In the United States, underrepresented minority (URM) groups, including African American or Black, Hispanic or Latino, and American Indian or Alaska Native populations, have experienced more barriers to substance use treatment engagement than their White counterparts [1]. These URM groups comprised only 15% of admissions to publicly funded substance use treatment programs in 2020, whereas White populations comprised 77% of admissions [2]. While the treatment gap is large for all, these gaps are greater for URM groups [3]. Among African American or Black and Hispanic or Latino populations diagnosed with a substance use disorder, 93% and 95%, respectively, did not receive treatment in 2021 [4]. Of the few URM groups who do access treatment, African American or Black and Hispanic or Latino populations are the largest groups and are less likely to complete treatment compared to White individuals [5-8].

American Indian or Alaska Native populations are overall at an increased risk of substance use disorder, particularly for alcohol use disorder [9]. These populations have historically been associated with excessive alcohol consumption, and yet a recent study using national survey data to compare alcohol use between American Indian or Alaska Native and non-Hispanic White individuals found limited differences for race and ethnicity, but the odds of binge drinking in male individuals were over twice those of their female counterparts [10,11]. It is notable that the number of American Indian or Alaska Native populations has consistently been too low to assess the treatment gap for those with a substance use disorder [4]. This statistical problem is likely due to the common research analytic strategy of collapsing demographic data into “more than one race,” thereby losing the potential insights into differences among those in this general category. Awareness of environmental risk factors (eg, for American Indian or Alaska Native populations, related to forced assimilation, such as living in geographically isolated communities without economic opportunities and the cross-generational transmission of the cumulative emotional and psychological trauma of cultural genocide) are essential to both prevention and treatment of substance use for all 3 URM groups that are the focus of this scoping review [10].

Disparities in treatment outcomes remain for these URM groups due in part to differences in social determinants and systemic racism as risk factors [1,12-14]. These differences are preventable as they are likely largely due to structural disadvantages that result in limited opportunities in health care, education, social context, economic stability, and the environment (ie, social determinants of health). To comprehensively address health equity—a state in which all people have a fair opportunity to attain their full health potential and well-being and no one is disadvantaged from doing so because of social position or any other socially defined circumstance [15]—avoidable inequalities and historical and contemporary injustices must be addressed to eliminate health care disparities.

Treatment intervention research, including substance use treatment research, has infrequently been conducted with people who identify as belonging to racial, ethnic, or both minority groups [16]. Indeed, a recent systematic review found that only 9 randomized controlled trials examined differences in substance use treatment outcomes by race or ethnicity for African American or Black and Hispanic or Latino populations, with even fewer reporting on differences in the baseline social determinants of health [17]. Thus, the state of treatment intervention research—particularly substance use treatment research—is a glaring example of limitations of opportunity for URM groups with respect to health care.

Advances in technology-based interventions (TBIs) show promise in reducing barriers and expanding access to evidence-based treatments [18]. However, there is a risk that TBIs may further exacerbate inequities by disproportionately benefiting people from higher socioeconomic status (SES) groups, who are largely White individuals [19]. Apprehension of this risk is warranted; throughout history in the United States, these URM groups have experienced violence and displacement due to settler colonialism (eg, for African American or Black individuals, capture from Africa and forced slavery and the US government’s social construction of Blackness as innately diseased due to presumed biological inferiority [15]; for Hispanic or Latino individuals, pre- and postmigration factors increasing the risk of intergenerational trauma, precarity due to legal status, discrimination, and anti-immigrant policies [20]), leading to the unequal distribution of resources and an overall lower SES [21,22]. However, in recent years, TBIs are increasingly being culturally adapted with the aim of increasing intervention effectiveness for specific URM groups [23-25]. In a systematic review of general technology use by URM groups, Montague and Perchonok [26] found that, while technology can be used to positively impact these communities, the technology must be culturally tailored to the specific URM group community to create positive behavior change. However, within substance use treatment, there has been limited research as to which specific characteristics of TBIs are most effective for URM groups [16].

### Objectives

This scoping review aimed to answer one primary question: does the use of TBIs in substance use treatment research promote health equity among people who identify as African American or Black, Hispanic or Latino, and American Indian or Alaska Native individuals? We operationally defined health equity as the inclusion of members of underrepresented groups (previously specified) in research on TBIs for substance use, as well as the extent to which the research is race and ethnicity conscious. Thus, the following questions helped establish an effective search strategy and results that responded to the primary research question: does research on TBIs for substance use treatment include people who identify as African American or Black, Hispanic or Latino, or American Indian or Alaska Native individuals? If the substance use research on TBIs does include people who identify as African American or Black, Hispanic or Latino, or American Indian or Alaska Native individuals, is it race and ethnicity conscious?

## Methods

### Protocol

This scoping review followed the PRISMA-ScR (Preferred Reporting Items for Systematic Reviews and Meta-Analyses extension for Scoping Reviews) [27]. The PRISMA-ScR checklist can be found in Multimedia Appendix 1. A summary of the methods is provided in this section, with a published protocol publicly available [28].

### Study Eligibility Criteria

#### Overview

The Joanna Briggs Institute—an organization that offers guidance for conducting systematic scoping reviews—recommends the use of the “PCC mnemonic” (population, concept, and context) for defining the focus of a review. The context for our review was substance use and disorder treatment research; the concept was TBIs; and the population were studies with samples that included at least 50% of participants who identified as African American or Black, Hispanic or Latino, or American Indian or Alaska Native individuals when combined [29].

#### Inclusion Criteria

The study inclusion criteria were US-based, English-language, peer-reviewed studies of TBIs for substance use treatment with a sample of at least 50% of participants identifying as African American or Black, Hispanic or Latino, or American Indian or Alaska Native individuals (combined) published between January 2000 and March 2021. The decision for the threshold of 50% was based on a review of evidence-based treatments for youth from ethnic minority groups. After the evidence-based criteria were met, the interventions were considered well established, probably efficacious, or possibly efficacious for youth from ethnic minority groups if supporting studies met one or more of several conditions, including whether at least 75% of participants in the study identified as being from ethnic minority groups [30]. By choosing a 50% threshold, we were more inclusive and likely netted more studies than we would have if we had chosen a 75% cutoff. We focused on this period due to a rising trend in the number of published studies starting in 2001 involving the design and development of technology-based behavior change interventions [31]. Study design criteria included qualitative, quantitative, and mixed methods studies.

#### Exclusion Criteria

We excluded the following manuscript types: protocol papers, descriptions of future work, reviews, commentaries, editorials, opinion pieces, student theses, conference abstracts, book chapters, and guidelines. Tobacco intervention studies were also excluded from this review. (see the work by Hichborn et al [32] for a separate scoping review on tobacco-focused TBI studies).

### Data Sources and Search Strategy

Before enlisting the help of research librarians, study team members conducted preliminary independent literature searches in PubMed and Google Scholar using search terms associated with the 3 domains of interest—TBIs, substance use treatment, and sample inclusion of members of URM groups—to identify whether there was indeed a gap in the literature to be filled by this scoping review. This step netted dozens of research studies that appeared to meet the inclusion criteria, confirming the gap in the literature. Following these exploratory efforts, a comprehensive search strategy was implemented using 5 electronic databases (MEDLINE, Scopus, Cochrane Library, CINAHL, and PsycINFO) to identify research that included substance use and disorder treatment interventions using TBIs [29]. The literature searches were conducted by 2 Dartmouth College reference librarians in consultation with the research team. The final electronic search strategy for MEDLINE can be found in Multimedia Appendix 2.

### Screening and Selection Procedure

Duplicate studies were removed in EndNote X9 (Clarivate Analytics), and the remaining studies were uploaded into the web-based review tool Rayyan (Rayyan Systems Inc) [33] for screening and selection. Studies were first screened at the title and abstract level and then selected for inclusion after a full-text review by a group of 8 reviewers (SKM, CAL-H, PRG, NA, ECS, EGH, KDB, and AMT) working in 3 small teams. Throughout the independent selection process, individual reviewers were blinded to each other’s decisions. The reviewers met regularly to discuss discrepancies and reach a consensus both within teams and as a full group.

### Data Extraction

The review team developed a standardized data extraction form in Microsoft Excel (Microsoft Corp) with predefined variables (Textbox 1). A standard operating procedure provided general extraction guidelines, variable definitions, and expected location of variables within the manuscripts. Before data collection, the reviewers piloted the extraction form with 2 studies and reviewed for reliability across team members. At this stage 2 more team members were added, and a 9-person review team continued to work in 3 small groups, with each team member individually performing extraction for a sample of articles within the pool assigned to their team. All studies had a second team member perform a quality assurance data validation check for accuracy, consistency, and thoroughness in the data extraction fields, mainly focusing on fields with the greater likelihood for inconsistency (eg, TBI).

**Data extraction variables.**

**Variable and description of variable**

- Author: last name of first author
- Year: year when the study was published
- Aim or aims: purpose of the technology-based intervention (TBI)
- Design: the study design
- Participant population (for whom): description of the target population that the study intended to enroll
- End-user involvement (with whom): description of methods used to involve or engage end users in the development, design, or adaptation of TBIs and opportunities for end users to provide feedback on TBIs to inform acceptability, among other things (intervention-focused outcomes)
- N: number of participants in the study
- Sample summary: racial and ethnic profile of the study participants
- Recruitment plan: description of how candidates were informed of and introduced to the study
- Retention plan: description of any efforts to retain participants in the research study
- Primary substance use target: substance use or substance use disorder targeted by the TBIs
- Context: description of intervention delivery setting and other relevant details (eg, urban, rural, remote, or in person)
- TBI: description of TBI and name (if named)
- Device: description of the device used to deliver the TBI
- Delivery mode: description of how the TBI was delivered to participants, including the frequency and duration
- Intervention components: description of interventional components combined with the TBI (eg, counseling and contingency management)
- Comparators: description of other interventions that the TBI was compared with
- Behavior change theory: description of behavior change theory or theories underlying the TBI
- Behavior change techniques: description of behavior change techniques that were used by the TBI
- Race and ethnicity consciousness: yes or no—specific race or ethnicity references in one or more sections of the manuscript (ie, introduction, methods, results, or discussion; see the Race and Ethnicity Consciousness section)

### Data Synthesis and Analysis

#### Organization

We organized netted studies by programs of research, which were defined as ≥2 studies that involved coauthorship networks and referenced the same TBI. Studies were classified as race or ethnicity conscious (yes or no) if the article specifically described research practices informed by at least one URM group in at least one section of the article (ie, title, introduction, method, results, or discussion; Textbox 2). Tables pertaining to race or ethnicity consciousness were populated with data relevant to the studies’ specific reference to the 3 URM groups across manuscript sections. The remaining extracted data served to represent the range of the studies and the nature of TBIs in their respective tables, as well as access and inclusion variables.

**Examples of race- and ethnicity-conscious practices in research.**

**Section of the article and example**

- Title: Tailoring an Alcohol Intervention for American Indian Alaska Native Women of Childbearing Age: Listening to the Community
- Introduction: providing epidemiological or other relevant information about one or more racial or ethnic groups in the literature review or using a theory that was described as one that may help address health disparities among racial and ethnic underrepresented groups. An example of this includes sociological trust theory—a bridge between a broad lens of culturally informed design and attention to trust or distrust.
- Methods: race-conscious methods may include references to recruitment or retention efforts aimed at racial or ethnic groups, such as cultural tailoring of materials, consideration of matching staff race and ethnicity to that of the sample participants (in telemedicine appointments and in the animations seen in virtual reality and computer games), or assessment for measurement equivalence. Race-conscious analytic plans may include conducting separate analyses for each race and ethnicity, focusing on within-group differences rather than race comparisons, using stratification methods that balance each race and ethnicity across treatment arms, or considering race and ethnicity in some other way in the plan for analyzing the data.
- Results: presenting findings in a way that highlights differences and similarities for members of different racial or ethnic groups.
- Discussion: interpreting findings for members of racial or ethnic groups by locating results in the context of other development or treatment literature.

#### Analysis

Included sources of evidence for scoping reviews are not critically appraised regarding methodological quality or risk of bias, making scoping reviews significantly different from systematic reviews. However, one of the PRISMA-ScR items includes an optional “critical appraisal of individual data sources” [27]. In this study, we aimed to critically appraise the research in terms of each study’s *potential* to help us better understand the impact of a TBI for members of URM groups. Our rationale for the critical appraisal was founded on the notion that promoting health equity includes highlighting all netted research and not solely focusing on the TBIs that have been found to be effective. To conduct the critical appraisal, we organized the studies by levels of inclusion of a single URM and race or ethnicity consciousness. A higher appraisal or confidence was assigned to studies explicit in presenting results clarifying study implications with samples that included ≥90% of members of a single URM group or included race-conscious results such that the findings were interpretable due to subgroup analyses based on race or ethnicity (eg, 30% African American or Black participants). A lower appraisal or confidence was assigned to those studies that included <90% of members of a single URM group *and* lacked race-conscious results.

The greater the representation of a single URM group in a sample, the more likely the findings were to have direct implications for that group regardless of whether the results were race conscious.

## Results

### Sources of Evidence

**Figure 1:**
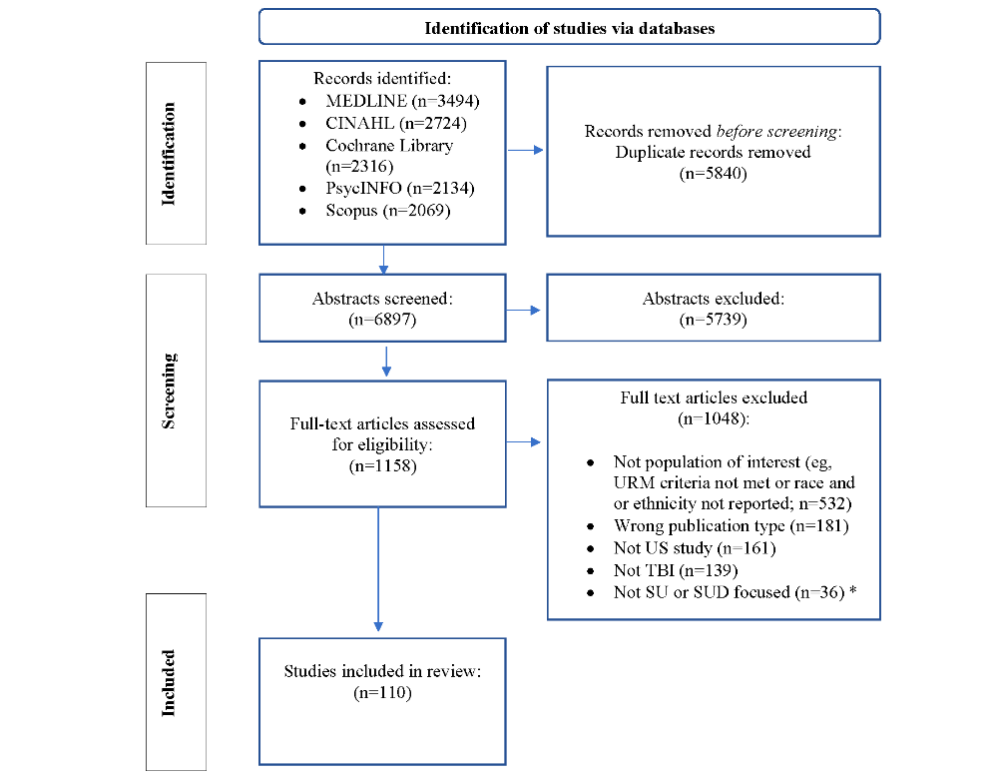
PRISMA-ScR (Preferred Reporting Items for Systematic Reviews and Meta-Analyses extension for Scoping Reviews) flow diagram [27]. SU/D: substance use or disorder; TBI: technology-based intervention; URM: underrepresented minority.

The systematic searches identified 6897 references (Figure 1 [27]). Title and abstract screening excluded 83.21% (5739/6897) of the references, and a further 90.5% (1048/1158) of the papers were excluded at full-text review, resulting in 110 studies meeting the inclusion criteria for this scoping review. Full-text review excluded 50.67% (531/1048) of the studies due to ineligibility based on demographic criteria or the absence of race or ethnicity data. Most of the studies (81/110, 73.6%) were published from 2014 onward.

### Scoping Review Dataset

The dataset was organized by programs of research (76/110, 69.1% of the studies) and independent studies, defined as studies without companion publications in the dataset (34/110, 30.9%; Table 1).

**Table 1:** Programs of research and independent studies (n=110).

Programs of research	Studies, n (%)
CEMA^a^	2 (1.8)
MOMENT^b^	2 (1.8)
e-SBI^c^ DrinkWise	2 (1.8)
TEXT^d^	2 (1.8)
Web-based SBIRT^e^	3 (2.7)
Web-based MI^f^	3 (2.7)
Tailored SMS text messaging	4 (3.6)
eCheckup	3 (2.7)
TES^g^	3 (2.7)
Theory-based SMS text messaging	3 (2.7)
EMA^h^	4 (3.6)
A-CHESS^i^	5 (4.5)
SaferTeens	6 (5.4)
Health Call	7 (6.3)
CBT4CBT^j^	10 (9)
MES^k^ or SBIRT	17 (15.5)
Independent studies	34 (30.9)

^a^CEMA: cellular ecological momentary assessment.

^b^MOMENT: Momentary Self-Monitoring and Feedback + Motivational Enhancement Therapy.

^c^e-SBI: electronic screening and brief intervention.

^d^TEXT: treatment extension by text.

^e^SBIRT: Screening, Brief Intervention and Referral to Treatment.

^f^MI: motivational interviewing.

^g^TES: Therapeutic Education System.

^h^EMA: ecological momentary assessment.

^i^A-CHESS: Addiction Comprehensive Health Enhancement Support System.

^j^CBT4CBT: Computer Based Training for Cognitive Behavioral Therapy.

^k^MES: Motivational Enhancement System.

### Study Characteristics

Study designs included 39.1% (43/110) randomized trials, 36.4% (40/110) feasibility studies, 7.3% (8/110) development studies, 6.4% (7/110) secondary analyses, 5.5% (6/110) assessments (ie, interactive voice response, ecological momentary assessment [EMA] solely, with TBI development planned), and 5.5% (6/110) miscellaneous study types. Of the eligible studies, 31.8% (35/110) examined TBIs combined with or integrated into treatment as usual (TAU), whereas 24.5% (27/110) examined TBIs combined with treatment or treatments that were novel to the existing treatment program (eg, motivational interviewing, contingency management [CM], and supportive messages). Of the 27 studies examining TBIs combined with novel treatments, 6 (22%) combined more than one novel treatment with the TBI (eg, naloxone training, HIV, and hepatitis C virus testing; motivational interviewing or transtheoretical model; reading scripture followed by song or relation breathing exercises; or cognitive behavioral therapy [CBT] or tai chi). Of the 110 included studies, 59 (53.6%) reported the use of a comparator to the TBI. Comparators included other TBIs (31/59, 53%); in-person, clinician-delivered versions of the TBI (10/59, 17%) and briefer versions of the targeted TBI (6/59, 10%); TAU (16/59, 27%); nonactive interventions (eg, reading a pamphlet; excluding TAU; 30/59, 51%); motivational interviewing (7/59, 12%); CBT or counseling (4/59, 7%); SMS text messaging or EMA (5/59, 8%); and others (16/59, 27%; eg, DVDs or videos, brochures and website resources, and assessment-only groups).

Beyond the sources of evidence, resultant scoping review dataset and study characteristics typically reported in scoping reviews, the following result subsections (ie, Access or Inclusion, Range and Nature of TBIs, Race and Ethnicity Consciousness, and the critical appraisal of the dataset) all derive from the primary research question and related subquestions developed based on the operationalization of health equity promotion.

### Access and Inclusion

#### Racial or Ethnic Sample Profiles (Analyzed Data)

In 78.2% (86/110) of the included studies, at least 50% of the participants were members of a single URM group: American Indian or Alaska Native (4/86, 5% of the studies) [34-37], Hispanic or Latino (15/86, 17% of the studies) [38-52], and African American or Black (67/86, 78% of the studies; Tables 2 and 3). While most of the studies on African American or Black individuals also included people who identified as Hispanic or Latino and vice versa, only 12.3% (13/106) of the studies with a majority of African American or Black or Hispanic or Latino individuals included people who identified as American Indian or Alaska Native individuals [46,47,49,53-62].

**Table 2:** Programs of research.

TBI^a^ program of research, study, and year		Substance use target	Inclusion
**Motivation Enhancement System^b^**			
	Ondersma et al [63], 2005	Drug use	97% African American or Black^c^
	Ondersma et al [64], 2007	Drug use	97% African American or Black^c^
	Ondersma et al [65], 2011	Drug use	100% African American or Black
	Ondersma et al [66], 2014	Drug use	90.6% African American or Black, 5.8% White, 3.6% other, and 1.5% Hispanic or Latino
	Tzilos et al [67], 2011	Alcohol	82% African American or Black, 16% White, and 3% Hispanic or Latino
	Ondersma et al [68], 2015	Alcohol	81.3% African American or Black^c^
	Pollick et al [69], 2015	Alcohol	100% African American or Black
	Ondersma et al [70], 2016	Alcohol	87% African American or Black
	Martino et al [71], 2018	ATOD^d^	66.7% African American or Black, 13.2% White, 14.8% Hispanic or Latino, and 5.2% other or multiple races
	Forray et al [72], 2019	ATOD	66.7% African American or Black, 13.2% White, 14.8% Hispanic or Latino, and 5.2% other or multiple races
	Loree et al [73], 2019	ATOD	66.7% African American or Black, 13.2% White, 14.8% Hispanic or Latino, and 5.2% other or multiple races
	Yonkers et al [74], 2020	ATOD	66.7% African American or Black, 13.2% White, 14.8% Hispanic or Latino, and 5.2% other or multiple races
	Ondersma et al [75], 2018	Drug use	73.2% African American or Black, 24.2% other or multiple races, and 2.6% White
	Braciszewski et al [53], 2018	AOD^e^	29% Hispanic or Latino, 24% African American or Black, 41% White, 18% American Indian or Alaska Native, 6% Hawaiian or Pacific Islander, 6% Asian or Pacific Islander, and 6% other
	Braciszewski et al [76], 2018	AOD	52% African American or Black, 27% White, 18% multiple races, and 3% other
	Tzilos Wernette et al [54], 2018	AOD	40% Hispanic or Latino, 33% African American or Black, 5% American Indian or Alaska Native, and 50% other or multiple races
	Ondersma et al [77], 2019	Cannabis	84.4% African American or Black and 1.2% Hispanic or Latino^c^
**CBT4CBT^f^**			
	Carroll et al [55], 2008	AOD	46% African American or Black, 34% White, 12.3% Hispanic or Latino, and 5.5% American Indian or Alaska Native
	Sugarman et al [56], 2010	AOD	46% African American or Black, 34% White, 12.3% Hispanic or Latino, and 5.5% American Indian or Alaska Native
	Kiluk et al [57], 2010	AOD	50% African American or Black, 36.5% White, 9.6% Hispanic or Latino, and 3.8% American Indian or Alaska Native
	Kiluk et al [58], 2011	AOD	50% African American or Black, 36.5% White, 9.6% Hispanic or Latino, and 3.8% American Indian or Alaska Native
	Kiluk et al [78], 2016	Alcohol	54.4% African American or Black, 11.8% Hispanic or Latino, 33.8% White, 7.4% Hispanic or Latino only, and 4.4% other or multiple races
	Kiluk et al [79], 2018	Alcohol	54.4% African American or Black, 11.8% Hispanic or Latino, 33.8% White, 7.4% Hispanic or Latino only, and 4.4% other or multiple races
	Kiluk et al [80], 2018	AOD	48.9% African American or Black, 34.3% White, 16.1% Hispanic or Latino (ethnicity), 8% Hispanic or Latino (only), and 8.7% other or multiple races
	Paris et al [38], 2018	AOD	100% Hispanic or Latino
	Roos et al [81], 2020	AOD	54.4% African American or Black, 11.8% Hispanic or Latino, 33.8% White, 7.4% Hispanic or Latino only, and 4.4% other or multiple races
	Jordan et al [82], 2021	AOD	100% African American or Black
**Health Call^g^**			
	Aharonovich et al [39], 2006	Alcohol	51.6% Hispanic or Latino, 37.8% African American or Black, 6.5% White, and 3.2% other
	Hasin et al [83], 2013	Alcohol	49% African American or Black, 45% Hispanic or Latino, and 6% other
	Hasin et al [84], 2014	Alcohol	61.5% African American or Black, 25.6% Hispanic or Latino, and 12.8% other
	Aharonovich et al [85], 2012	AOD	63.6% African American or Black, 21.2% Hispanic or Latino, and 6.1% White
	Aharonovich et al [86], 2017	AOD	55% African American or Black, 25.8% Hispanic or Latino, and 19.17% other
	Aharonovich et al [87], 2017	AOD	78.72% African American or Black and 17.02% Hispanic or Latino
	Thompson et al [59], 2020	Alcohol and cannabis	65% African American or Black, 47.5% Hispanic or Latino, 15% American Indian or Alaska Native, 10% White, 5% Native Hawaiian or Pacific Islander, and 2.5% Asian
**SaferTeen^h^**			
	Cunningham et al [88], 2009	Alcohol	55% African American or Black, 37.2% White, 6.2% Hispanic or Latino, and 7.8% other
	Walton et al [89], 2010	Alcohol	55.9% African American or Black, 39.1% White, and 6.5% Hispanic or Latino
	Walton et al [90], 2013	Cannabis and AOD	60.7% African American or Black and 11% Hispanic or Latino
	Blow et al [91], 2017	AOD	52.2% African American or Black, 39.2% White, 8.6% other, and 6% Hispanic or Latino
	Waller et al [92], 2019	Cannabis	54% African American or Black^c^
	Drislane et al [93], 2020	Alcohol and cannabis	52.2% African American or Black, 39.2% White, 8.6% other, and 6% Hispanic or Latino
**A-CHESS and CASA-CHESS^i^**			
	Dennis et al [94], 2015	AOD	48% African American or Black, 21% White, 7% Hispanic or Latino, and 24% multiple races
	Scott et al [95], 2013	—^j^	85% African American or Black, 6% Hispanic or Latino, 6% White, and 3% other
	Muroff et al [40], 2017	AOD	96.2% Hispanic or Latino and 3.8% African American or Black (Spanish speaking)
	Muroff et al [41], 2019	AOD	96.2% Hispanic or Latino and 3.8% African American or Black (Spanish speaking)
	Scott et al [96], 2020	AOD	70% African American or Black, 20% White, 6% Hispanic or Latino, and 4% other or multiple races
**EMA or electronic diary^k^**			
	Preston et al [97], 2016	AOD	68% African American or Black and 43% White
	Preston et al [98], 2017	Opioids and cocaine	64.8% African American or Black and 34% White
	Preston et al [99], 2018	Opioids and cocaine	64.8% African American or Black and 34% White
	Preston et al [100], 2018	Opioids and cocaine	64.8% African American or Black and 34% White
**Theory-based SMS text messaging^l^**			
	Reback et al [101], 2012	Methamphetamines	38.5% Hispanic or Latino, 21.2% African American or Black, 34.6% White, and 5.8% other or multiple races
	Reback et al [102], 2015	Methamphetamines	38.5% Hispanic or Latino, 21.2% African American or Black, 34.6% White, and 5.8% other or multiple races
	Reback et al [103], 2019	Methamphetamines	43.7% African American or Black, 25.2% Hispanic or Latino, 19.6% White, and 11.5% other or multiple races
**TES^m^**			
	Brooks et al [104], 2010	AOD	100% African American or Black
	Campbell et al [34], 2015	AOD	100% American Indian or Alaska Native
	Budney et al [105], 2015	Cannabis	59% African American or Black and 41% White
**Women, Infant, and Children eCheck-up^n^**			
	Delrahim-Howlett et al [106], 2011	Alcohol	44% Hispanic or Latino, 34% White, 8% African American or Black, 8%, other and 14% other or multiple races
	Gorman et al [35], 2013	Alcohol	100% American Indian or Alaska Native
	Montag et al [36], 2017	Alcohol	84.2% American Indian or Alaska Native^c^
**Interactive Mobile Text Message Reminder System^o^**			
	Muench et al [60], 2013	AOD	50% African American or Black, 36.5% White, 9.6% Hispanic or Latino, and 3.8% American Indian or Alaska Native
	Tofighi et al [107], 2016	Opioids	39% African American or Black, 36% White, 22% Hispanic or Latino, and 3% other
	Tofighi et al [108], 2017	Opioids	40% African American or Black, 18% Hispanic or Latino, 41% White, and 1% Asian
**Telemedicine- or telephone-based opioid treatment—COVID-19**			
	Tofighi et al [109], 2022	Opioids	34.6% Hispanic or Latino, 34.2% White (non–Hispanic or Latino), 19.2% African American or Black (non–Hispanic or Latino), and 15.4% other
**Culturally relevant, web-based motivational interviewing^p^**			
	Osilla et al [42], 2012	Alcohol	100% Hispanic or Latino
	Osilla et al [110], 2015	Alcohol	40% Hispanic or Latino, 39% African American or Black, 35% White, and 7% Asian or Pacific Islander
	Kennedy et al [111], 2018	AOD	56% African American or Black, 12% White (non–Hispanic or Latino), 15% Hispanic or Latino, and 17% other or multiple races
**Unnamed SBI^q^**			
	Dawson Rose et al [112], 2015	ATOD	42.7% African American or Black and 15.6% Hispanic or Latino
	Dawson Rose et al [113], 2017	ATOD	40% African American or Black, 30% White, 17% Hispanic or Latino, and 13% other
	Saberi et al [114], 2020	ATOD	35.6% Hispanic or Latino, 29.7% African American or Black, 13.9% White, 17.8% other or non–Hispanic or Latino
**Video+ and Treatment Extension by Text^r^**			
	Ingersoll et al [61], 2011	Cocaine	81.5% African American or Black, 13% White, 3.7% other, 1.9% American Indian or Alaska Native, and 1.9% Hispanic or Latino
	Ingersoll et al [115], 2015	Cocaine	65.1% African American or Black, 28.6% White, and 6.4% other
**e-SBI^s^ DrinkWise**			
	Nayak et al [43], 2014	Alcohol	62% Hispanic or Latino, 16% African American or Black, 17% other or multiple races, and 5% White
	Nayak et al [44], 2019	Alcohol	75% Hispanic or Latino, 17% White, 5% African American or Black, and 3% other
**MOMENT-MET+motivation and feedback^t^**			
	Shrier et al [116], 2014	Cannabis	44% African American or Black and 37% Hispanic or Latino^c^
	Shrier et al [117], 2018	Cannabis	47% African American or Black (non–Hispanic or Latino), 31% Hispanic or Latino, 11.4% White (non–Hispanic or Latino), and 10% multiple races or other (non−Hispanic or Latino)
**Cellular ecological momentary assessment^u^**			
	Comulada et al [45], 2015	AOD	93% Hispanic or Latino, 3.5% Asian or Pacific Islander, and 3.5% other
	Swendeman et al [118], 2021	AOD	55% African American or Black, 30% Hispanic or Latino, 5% White, and 5% Asian or Pacific Islander

^a^TBI: technology-based intervention.

^b^Motivation Enhancement System (electronic screening, brief intervention, and referral to treatment [eSBIRT]): a single-session eSBIRT. Assessment—questions presented one at a time using relevant graphics and a 3D cartoon character (program narrator and guide) capable of ≥50 specific animated actions (eg, smiling and expressing concern). Headphone use assures privacy. Intervention—three components based on motivational interviewing (MI) and brief intervention principles: (1) feedback regarding the negative consequences of reported drug use, self-reported readiness to change, and normative feedback (eg, drug use compared to drug use of all American adult women); (2) pros and cons of drug use and related change (participant chooses from a list of options); and (3) a summary and query regarding the participant’s interest in change, followed by optional goal setting regarding drug use. Throughout the intervention, the narrator reflects back the participant’s answers and helps establish an atmosphere that is as similar as possible to an in-person MI session.

^c^No further demographic information included on the racial and ethnic makeup of the sample.

^d^ATOD: alcohol, tobacco, or drug use.

^e^AOD: alcohol or drug use.

^f^CBT4CBT: Computer-Based Training for Cognitive Behavioral Therapy (computer-based intervention [CBI]). A computer-based version of cognitive behavioral therapy used in conjunction with standard clinical care for current substance users. Six modules and follow-up assignments focus on key concepts in substance use: (1) understanding and changing patterns of substance use, (2) coping with craving, (3) refusing offers of drugs and alcohol, (4) problem-solving skills, (5) identifying and changing thoughts about drugs and alcohol, and (6) improving decision-making skills. The first module provided a brief explanation of how to use and navigate the program; following completion of the first module, participants could choose to access the modules in any order that they preferred and repeat any section or module as many times as they wished. The multimedia presentation (graphic illustrations, videotaped examples, verbal instructions and audio voice-overs, interactive assessments, and practice exercises), which are based on elementary-level computer learning games, requires no previous experience with computers.

^g^Health Call (interactive voice response [IVR] and ecological momentary assessment intervention or SMS text messaging): automated daily telephone self-monitoring system for drinking based on IVR. Generically, IVR is a telephone-based procedure that allows individuals to interact with recorded questions and statements.

^h^SaferTeen (CBI): interactive multimedia computer program viewed on tablet laptops, with touch screens and audio delivered through headphones to ensure privacy. It was narrated and cartoon style, and participants could choose a gender-, race-, and age-appropriate “buddy” to “hang out” with throughout the session. The buddy guides participants through the intervention, including review of tailored feedback based on survey responses, identifying reasons to stay away from drinking and fighting, and role-play scenarios chosen by the computer based on reported risk behaviors. During the scenarios, participants had to interact with peers and make behavioral choices. Feedback was provided on these behavioral choices by the buddy, with possible consequences highlighted and the best possible outcome demonstrated by the characters.

^i^A-CHESS and CASA-CHESS: Addiction Comprehensive Health Enhancement Support System and its translation or adaptation for Spanish-speaking Latinos (IVR or ecological momentary assessment intervention or SMS text messaging). Ecological momentary assessments and recovery support ecological momentary interventions (EMIs) via smartphones. A-CHESS recorded the date, time, location, and responses to all ecological momentary assessments. The EMIs that adolescents could access at any time included (1) recovery support (discussion groups, support team, reaching out to others via SMS text message, listening to recovery stories, web-based self-help meetings, in-person self-help meeting locator, and linking to a sponsor), (2) relaxation (guided relaxation, playing games, listening to music, learning or reading, and physical exercise), (3) recovery motivation (motivational SMS text messaging, recovery words, recovery profiles, and pictures), and (4) social networking (Facebook and contacting friends).

^j^Not applicable.

^k^EMA: ecological momentary assessment. IVR or EMA intervention or SMS text messaging. Transactional electronic diary (EMA on PDA). Smartphones programmed with electronic diary software to prompt participants 3 times per day to answer a series of questions. The timing of the prompts was random but constrained by the participant’s self-reported typical waking hours for each day of the week. Participants also initiated entries whenever they experienced a stressful event (SE). Participants initiated an SE entry any time they felt “more stressed, overwhelmed, or anxious than usual” and checked whichever applied when they initiated an entry (multiple responses were possible). Participants categorized the severity of each SE, rated the associated feeling, and indicated the cause of the feeling by selecting from a list (a “fill in the blank” option was also available). In each of these SE entries, participants rated cravings for opioids and cocaine and reported whom they were with, what they were doing, and the type of EMA entry (SE entry vs randomly prompted entry; 2017-2018).

^l^Theory-based SMS text messaging (IVR or EMA intervention or SMS text messaging): real-time SMS text messaging intervention (400 SMS text messages). Prewritten SMS text messages were categorized by their theoretical base and by participant profile (eg, HIV status or whether a participant went online to “hook up”). On the basis of their responses to the 5 brief questions regarding their methamphetamine use administered at baseline as well as their SMS text messaging conversations, participants received messages that fit their profile.

^m^TES: Therapeutic Education Systems (CBI). Interactive, web-based program theoretically grounded in the evidence-based Community Reinforcement Approach to behavior therapy. It comprised interactive multimedia modules, including those focused on cognitive behavioral skill training (eg, effective strategies for refusing drugs and managing thoughts about drug use). TES also included modules to prevent HIV, hepatitis, and sexually transmitted infections. Additional modules taught skills to improve psychosocial functioning (eg, family or social relationships and managing negative moods). TES is a self-directed program that includes a module teaching patients how to use the system and a “customization program” to build an individualized treatment plan for patients.

^n^Women, Infant, and Children (WIC) eCheck-up (eSBIRT): adapted from eCHECKUP TO GO, a brief assessment with motivational feedback tailored to college students. Tailored reading or comprehension level for WIC clients; visual graphics modified for this population. Changes to measurement components incorporated established and validated methods of assessing for alcohol use in women of childbearing potential and feedback tailored to include either general information about fetal alcohol syndrome or personalized information about participants’ alcohol use, associated health risks, and health risks associated with alcohol use during pregnancy.

^o^Interactive Mobile Text Message Reminder System (IVR or EMA intervention or SMS text messaging): the SMS text message reminder software notified patients 7, 4, and 1 days before their scheduled office based opioid treatment program appointments. Following enrollment, 1 message was sent to inform participants about terminating SMS text message reminders. Patient privacy was ensured by excluding any patient health information and clinic identifiers, such as “buprenorphine clinic,” within the SMS text message content.

^p^Culturally relevant, web-based MI (CBI): web-based MI with feedback tailored to baseline survey responses about drinking behavior and perceptions. Baseline survey responses populated the web-based MI content: (1) how their drinking and guesses of others’ drinking compared to those of other men or women their age in the United States, (2) their positive beliefs about drinking, (3) their negative consequences from drinking, and (4) strategies for avoiding consequences in the future.

^q^Unnamed screening and brief intervention (eSBIRT): self-administered SBI for substance use embedded in a web-based personal health record linked to the clinic’s electronic medical record (in English and Spanish) accessible via the internet. The SBI was tailored to patients’ substance dependence risk scores on the Alcohol, Smoking, and Substance Involvement Screening Test. No- or low-risk participants received positive feedback and behavior maintenance support. Moderate-risk participants received a brief interactive web-based intervention and links to substance use websites and patient resources. High-risk participants were referred to treatment (Saberi et al [114], development for ART adherence among youth living with HIV videoconferencing [video]).

^r^Video+ (video): 4 accurate videos with at least some personal narrative in addition to didactic information presented by diverse peer role models and medical experts. The videos addressed either crack cocaine use or drug use generally, HIV treatment, or both (only the study by Ingersoll et al [61]); Treatment Extension by Text (IVR or EMA or SMS text messaging): bidirectional SMS text messaging system using EMA that sends messages to and receives and interprets messages from participants, enabling the system to send an appropriate intervention response. The automated system sent daily queries of medication dosing, mood, and substance use (only the study by Ingersoll et al [115]; platform changes within program of research).

^s^e-SBI: electronic screening and brief intervention.

^t^MOMENT-MET+motivation and feedback (IVR or EMA intervention or SMS text messaging): brief motivational therapy administered by a counselor in the clinic with mobile self-monitoring and responsive messaging via an electronic mobile device to extend treatment into the natural environment and everyday life.

^u^Cellular ecological momentary assessment (IVR or EMA intervention or SMS text messaging): SMS text messaging–based EMA focused on (1) alcohol or drug use, (2) quantity used, (3) antecedents, (4) intensity of craving, (5) engagement in risky behaviors, and (6) research participants’ activities and location.

**Table 3:** Independent studies.

Platform and study		Substance use targets	Inclusion	TBI^a^
**Video, videoconferencing, or telepsychiatry**				
	Millery et al [46], 2002	Opioids and cocaine	58% Hispanic or Latino, 33% African American or Black, 5% White, 3% Asian, American Indian or Alaska Native, and 1% other	Video resource intervention
	Frueh et al [119], 2005	Alcohol	83% African American or Black and 17% White	Telepsychiatry or videoconference
	Wechsberg et al [120], 2011	AOD^b^	100% African American or Black	Pregnant women’s WHC^c^ intervention; Microsoft PowerPoint (Microsoft Corp) presentation via video
	Brusoski and Rosen [121], 2015	Opioids	100% African American or Black	Healthy Living Intervention—video chat
	Welsh [122], 2016	Opioids	55% African American or Black and 45% White	Cellphone or computer pictures or video for pill counts for OUD^d^
	Jaconis et al [123], 2017	AOD	100% African American or Black	Telehealth or videoconference—“COPE”^e^
	Palfai et al [124], 2019	Alcohol	80% African American or Black and 20% Hispanic or Latino	Development study plan to use videoconferencing
	Castillo et al [125], 2020	Opioids	38% Hispanic or Latino, 43% White, and 19% African American or Black (non–Hispanic or Latino)	Tele-MOUD^f^
	Legha et al [37], 2020	AOD	100% American Indian or Alaska Native	Telepsychiatry or video
	Mehtani et al [126], 2021	Opioids	67% African American or Black, 8% Hispanic or Latino, and 33% White	ATP^g^
	O’Gurek [127], 2021	Opioids	40% African American or Black, 36% White, 22% Hispanic or Latino, and 1.9% other	Telehealth or video—COVID-19
**VR^h^ or avatar**				
	Saladin et al [128], 2006	Cocaine (“crack”)	91% African American or Black	VR environment—cocaine (“crack house” simulation)
	Gordon et al [129], 2017	AOD	59.3% African American or Black, 39% White, and 1.7% other	AAT^i^
**EMA^j^**				
	Freedman et al [130], 2006	Cocaine (“crack”)	93% African American or Black and 7% White	EMA via cell phone with homeless adults addicted to crack cocaine in an IOP^k^
	Linas et al [131], 2015	Opioids and cocaine	90% African American or Black^I^	Unnamed EMA for drug use
	Yang et al [132], 2015	Alcohol	100% African American or Black	EMA for AA^l^ men who have sex with men
	Sanjuan et al [47], 2019	AOD	66.7% Hispanic or Latino, 78.8% White, 21.2% American Indian or Alaska Native, and 3% African American or Black	EMA for treatment-engaged pregnant women with SUDs^m^
**SMS text messaging**				
	Trudeau et al [48], 2012	AOD	62.5% Hispanic or Latino (White or African American or Black), 29.2% White, and 8.3% African American or Black (non–Hispanic or Latino)	Web-based relapse prevention for adolescents (SMS text messaging)
	Buu et al [133], 2017	AOD	60% African American or Black and 20% White	IVR^n^ or SMS text messaging—daily or weekly
	Suffoletto et al [134], 2018	Alcohol	54% African American or Black and 46% White	TRAC2^o^
	Tolou-Shams et al [49], 2019	AOD	60% Hispanic or Latino, 33.33% African American or Black, 7% American Indian or Alaska Native, 13.33% Asian, 13.33% multiple races, and 27% other	SMS text message development for youth on probation and their caregivers
	Moore et al [135], 2019	AOD	53% African American or Black (non–Hispanic or Latino), 31% Hispanic or Latino, and 16% other (non–Hispanic or Latino)	CBT^p^ or tai chi protocol reinforced with SMS text messaging
	Glasner et al [136], 2020	AOD	60% African American or Black, 25.7% Hispanic or Latino, and 14.3% White	SMS text messaging or CBT intervention (ALC-TXT-CBT^q^)
**eSBIRT^r^ or eSBI^s^**				
	Mullen et al [50], 2015	Alcohol	75% Hispanic or Latino, 94% White, 2% African American or Black, and 4% multiple races or other	MATTERS^t^—eSBIRT with DUI^u^ offenders
	Burner et al [51], 2020	Alcohol	88% Hispanic or Latino	MROAD^v^—SMS text messaging adaptation in Spanish or SBIRT^w^
**CBI^x^**				
	Spohr et al [137], 2015	AOD	65.8% African American or Black and 27.6% White	MAPIT^y^—CBI for probationers+voluntary SMS text message or email reminders
	Steers et al [138], 2016	Alcohol	42% Hispanic or Latino, 46% White, 14% African American or Black, 20% other, and 4% multiethnic	PNF^z^—web-based intervention
	Aronson et al [52], 2017	Drug use	55% Hispanic or Latino (White), 10% White (non–Hispanic or Latino), 23% African American or Black (non–Hispanic or Latino), and 6% African American or Black (Hispanic or Latino)	MIK^aa^—people with disabilities or people with HIV (web-based CBI)
	Ditre et al [139], 2019	Opioids and tobacco	47.1% African American or Black, 41.2% White, 11.8% Hispanic or Latino, and 11.8% Asian	PFI^ab^ for people living with HIV—CBI
	Gryczynski et al [140], 2021	AOD	84% African American or Black, 13% multiple races, 6% Hispanic or Latino (any race), and 2% White	Computer-delivered behavioral intervention (CBI)
**Pill dispenser+CM^ac^**				
	Moore et al [141], 2015	AOD	60% African American or Black, 40% White, 20% Hispanic or Latino, and 80% non–Hispanic or Latino	CARE^ad^ electronic pill dispenser (ART^ae^), CM via electronically loaded study debit cards, and CBT for HIV (phone based)
**mCM^af^**				
	Beckham et al [142], 2018	Cannabis and tobacco	100% African American or Black	ART—ART combines mCM, telephone CBT, and a telehealth clinic for NRT^ag^
**Mobile app**				
	Babson et al [143], 2015	Cannabis	50% African American or Black, 50% White, and 25% Hispanic or Latino	CBT-I^ah^ iOS Coach (mobile app)
**Email**				
	Alemi et al [62], 2010	AOD	67% African American or Black (non–Hispanic or Latino), 11% White (non–Hispanic or Latino), and 15% American Indian or Alaska Native	MI^ai^ counseling (via email)

^a^TBI: technology-based intervention.

^b^AOD: alcohol or drug use.

^c^WHC: Women’s Health CoOp.

^d^OUD: opioid use disorder.

^e^COPE: Concurrent Treatment of Posttraumatic Stress Disorder and Substance Use Exposure Using Prolonged Exposure.

^f^MOUD: medication for opioid use disorder.

^g^ATP: Addiction Telehealth Program.

^h^VR: virtual reality.

^i^AAT: avatar-assisted therapy.

^j^EMA: ecological momentary assessment.

^k^IOP: intensive outpatient program.

^l^AA: African American.

^m^SUD: substance use disorder.

^n^IVR: interactive voice response.

^o^TRAC2: Texting to Reduce Alcohol Consumption.

^p^CBT: cognitive behavioral therapy.

^q^ALC-TXT-CBT: cognitive behavioral therapeutic texting intervention for targeting alcohol use.

^r^eSBIRT: electronic screening, brief intervention, and referral to treatment.

^s^eSBI: electronic screening brief intervention.

^t^MATTERS: Motivational Alcohol Treatments to Enhance Roadway Safety.

^u^DUI: driving under the influence.

^v^MROAD: Mobilizing to Reduce Overuse of Alcohol in the emergency department.

^w^SBIRT: screening, brief intervention, and referral to treatment.

^x^CBI: computer-based intervention.

^y^MAPIT: Motivational Assessment Program to Initiate Treatment.

^z^PNF: personalized normative feedback.

^aa^MIK: Mobile Intervention Kit.

^ab^PFI: personalized feedback intervention.

^ac^CM: contingency management.

^ad^CARE: Centralized Off-Site Adherence Enhancement.

^ae^ART: antiretroviral therapy.

^af^mCM: mobile contingency management.

^ag^NRT: nicotine replacement therapy.

^ah^CBT-I: cognitive behavioral therapy for insomnia.

^ai^MI: motivational interviewing.

#### Recruitment and Retention Efforts

A total of 65.5% (72/110) of the included studies recruited participants from medical centers (43/72, 60%), substance use treatment settings (26/72, 36%), or combined and integrated medical and substance use treatment programs in academic settings (3/72, 4%). A handful of studies recruited from social service programs (6/110, 5.5%; eg, Special Supplemental Nutrition Program for Women, Infants, and Children; foster care transition services; and crisis shelters), justice settings (5/110, 4.5%; eg, courts and programs for driving under the influence), and community settings (2/110, 1.8%; eg, church and syringe service programs). A total of 16.4% (18/110) of the studies recruited from more than one setting, and in other studies, recruitment was not described (2/110, 1.8%) or did not apply (4/110, 3.6%; eg, secondary analyses). Retention efforts included compensation for study participation, the device delivering the TBI being given to participants to keep after study completion, and a combination of these strategies. Some studies did not describe retention efforts, and in many instances, they were not applicable (ie, single-session TBI).

#### Device or Internet Access Requirements

Most studies (64/110, 58.2%) did not require access to the internet or a device for the TBIs. Among the studies that required internet or a device, 24.5% (27/110) provided devices either to all study participants or to those in need, and 18.2% (20/110) required participants to own or have access to a device to be eligible to take part in the study. The TBIs in 39.1% (43/110) of the studies were delivered on-site and required internet access, whereas in 25.5% (28/110) of the studies, they were delivered in remote settings and also required internet access. Geographically, the TBIs in 51.8% (57/110) of the studies were delivered in urban settings; however, 44.5% (49/110) of the studies did not clearly report the geographic setting. A total of 49.1% (54/110) of the studies described the involvement of potential end users, defined as individuals or groups for which the technology was developed, in the formative development or adaptation of a TBI using one or more of the following: interviews (19/54, 35%) [34,35,48,49,52, 54,59,69,76,82,84,87,108,114,118,119,121,124,132], focus groups (15/54, 28%) [35,36,42,45,53,60,82,84,88,110, 120,135,136], feedback sessions or gathering (11/54, 20%) [60,61,63-65,67,68,70,75,128,130], surveys (13/54, 24%) [48,50,53,70,76,82,94,107,116-118,122,141,143], usability testing (6/54, 11%) [47,54,58,108,110,112], and review by native speakers of the new adaptation (3/54, 6%) [40,41,115]. Many studies detailed the use of more than one method to involve potential end users.

#### End-User Groups

The dataset can be categorized into 11 end-user groups: childbearing-aged, pregnant, or perinatal women (22/110, 20%) [35,36,43,44,47,54,63-75,77,106,120], people living with HIV or AIDS (18/110, 16.4%) [39,61,83-87,112-115, 118,122,124,131,135,136,139,141], people in substance use treatment (17/110, 15.5%) [34,37,60,96-100,104,107,108, 119,121,122,127], people who inject drugs (2/110, 1.8%) [52,125], people seeking substance use treatment (15/110, 13.6%) [38,40,41,55-58,78-82,105,109,129], adolescents or youth (12/110, 10.9%) [45,48,88-90,94,116,117,133,140], youth exiting foster care (2/110, 1.8%) [53,75], people who were justice or court involved (6/110, 5.5%) [42,49,50,95,110,137], people being treated in hospital (detoxification) or emergency departments (6/110, 5.5%) [46,91-93,134], people who were unhoused (4/110, 3.6%) [59,111,126,130], men who have sex with men (5/110, 4.5%) [101-103,118,132], veterans (2/110, 1.8%) [123,143], non–treatment-seeking adults (1/110, 0.9%) [128], community recruits (2/110, 1.8%) [62,142], and college students (1/110, 0.9%) [138].

#### Social Determinants of Health

When conducting a parallel scoping review that focused on tobacco use [32], we observed patterns of reported environmental conditions indicative of low SES (eg, being unemployed, having a low or poverty-level income, and being uninsured) among study participants. On the basis of this observation, we explored the dataset for this review and found similar patterns of low income (20/110, 18.2%; eg, 81% of the participants had a barely or totally inadequate income) [116], unemployment (31/110, 28.2%; eg, “18 subjects and only one was employed”) [118], lack of health insurance or state insurance (6/110, 5.5%; eg, 86% of the participants were on Medicaid or uninsured) [108], homelessness (4/110, 3.6%; eg, homeless adult patients in treatment [126]), or low educational attainment (8/110, 7.3%; eg, average of 12 years of education) [143]. Another group of studies in this review included those on adolescents that reported no SES (9/110, 8.2%). Notably, many of the people in these samples fit multiple low-SES categories, but the studies were categorized without duplication based on the most salient SES variable in the study demographics (eg, ≥50% of the sample). Finally, 15.5% (17/110) of the studies, which were focused on adults, did not report SES, and another 10.9% (12/110) of the studies appeared to have higher average levels of SES (eg, more than half of the sample were employed). Thus, aside from studies on adolescents and other studies that did not report SES, as well as a small group that had participants of a slightly higher SES, the study samples of the remaining 65% of the studies represented people with a very low SES.

### Range and Nature of TBIs

#### Platform

Almost half (58/110, 52.7%) of the studies used computer-based intervention platforms, including electronic screening, brief interventions, and referrals to treatment. More specifically, 22.7% (25/110) of the studies used electronic screening, brief interventions, and referrals to treatment. Computer-based interventions were delivered via tablets and computers both with and without internet. One-third (37/110, 33.6%) of the dataset represented interactive voice response, EMAs or recovery support EMA interventions, or SMS text messaging intervention platforms delivered both with and without internet via mobile phones. The last large platform category was video, videoconferencing, or telepsychiatry (14/110, 12.7%). The remaining platforms included virtual reality or avatars (2/110, 1.8%) delivered via a computer, a pill dispenser+CM (1/110, 0.9%), a mobile app (1/110, 0.9%), mobile CM (1/110, 0.9%), and an email intervention delivered via a computer (1/110, 0.9%).

#### Substance Use Categories

Study-labeled substance use categories included alcohol or drug use or alcohol, tobacco, or drug use (45/110, 40.9%); alcohol only (27/110, 24.5%); and drug use only (6/110, 5.5%). Of the 110 studies, there were 8 (7.3%) opioid-focused studies [107-109,121,122,125-127], 5 (4.5%) that focused on opioids and cocaine [46,98-100,131], and 1 (0.9%) on opioids and tobacco [139]. A total of 5.5% (6/110) of the studies were cannabis focused [77,92,105,116,117,143], and an additional 4 were cannabis or alcohol focused (n=2, 50%) [59,93], cannabis or alcohol or drug use focused (n=1, 25%) [90], and cannabis or tobacco focused (n=1, 25%) [142]. Finally, 3.6% (4/110) of the studies focused on cocaine exclusively [61,115,128,130], and 2.7% (3/110) of the studies focused on methamphetamines [101-103]. It is important to note that the use of the study authors’ substance use labels resulted in an overlap of categories (eg, alcohol or drug use overlaps with drug use and cannabis, opioids, cocaine, and methamphetamines; Tables 2 and 3).

#### Behavior Change Theories and Techniques

One of the extraction variables for the review was behavior change theories and techniques. Of the 110 analyzed studies, 81 (73.6%) were based on *at least one* underlying behavior change theory, whereas 29 (26.4%) did not report an underlying theory or it was not applicable (ie, EMA studies). The top 6 most frequently referenced underlying theories were CBT (41/81, 51%); (20/81, 25%) screening, brief interventions, and referrals to treatment (10/81, 12%); self-determination theory (5/81, 6%); social cognitive theory (4/81, 5%); and the health belief model (4/81, 5%). A total of 78.2% (86/110) of the studies reported using *at least one* behavior change technique in their TBIs. Studies reported including the following behavior change techniques: feedback (33/86, 38%), goal setting (19/86, 23%), skill training (18/86, 21%), reminders (17/86, 20%), self-monitoring (16/86, 19%), EMA (14/86, 16%), and social support (7/86, 8%).

#### Access or Delivery Mode and Frequency

The TBIs were most commonly accessed or delivered independently, with the interventions in 65 studies either being self-initiated (n=45, 69%) or using software-automated prompts (n=20, 31%). A subset of the TBIs were delivered with the support of another individual, including service providers (15/110, 13.6% of the studies) and research team members (7/110, 6.4% of the studies). The interventions in 11.8% (13/110) of the studies were delivered via a hybrid model, combining self-initiated with personnel support. Delivery frequency of the included TBIs was predominantly in a single session (with or without booster sessions; 30/110, 27.3% of the studies) or daily for a limited period (25/110, 22.7% of the studies).

#### Programs of Research

Programs of research (16/16, 100%) ranged from a minimum of 2 to a maximum of 17 studies and, in some cases, spanned decades. Given space constraints, we feature the 2 largest programs (Motivation Enhancement System [MES] and Computer-Based Training for Cognitive Behavioral Therapy [CBT4CBT]) and encourage readers to explore other programs and independent studies (Tables 2 and 3) to obtain a sense for how a program of research might evolve to promote health equity. The largest program was MES, a computer-delivered, single-session, self-initiated brief intervention originated in a publication in 2005 [63]. This early study provided the material for an additional 14.5% (16/110) of the studies in the dataset, all of which explored MES’s utility primarily among perinatal or childbearing-aged women who identified as African American or Black individuals. MES was explored for utility among young adults who had recently exited foster care (ie, interactive health lifestyle preparation) and for utility with perinatal or childbearing-aged women who had different substance use profiles. The single-session nature of the tool also evolved to be combined with SMS text messaging within the program of research.

The second largest program (10/110, 9.1% of the studies) was CBT4CBT, a self-initiated, multi-session computer- or tablet-based version of CBT used in conjunction with standard clinical care for people currently using substances. The original, small-efficacy study comparing CBT4CBT to TAU [55] was referenced as source material in an additional 8.2% (9/110) of the studies in the dataset, including exploration of the TBI in small randomized trials exploring mechanisms as well as a culminating randomized controlled trial evaluating the efficacy and safety of CBT4CBT as a stand-alone treatment. The largest proportion of the samples in the first 70% (7/10) of the studies within this program comprised people who identified as African American or Black; however, each sample also included people who identified as Hispanic or Latino and some who identified as American Indian or Alaska Native. A total of 20% (2/10) of the most recent studies in this program of research included clear examples of work to promote health equity. Paris et al [38] described the primary outcomes of a randomized trial comparing a culturally adapted version of CBT4CBT (CBT4CBT-Spanish) to standard mental health and addiction treatment in treatment-seeking Latino adults, and Jordan et al [82] assessed the feasibility outcomes of a clinical trial for African American or Black adults with substance use disorder in a novel setting—a Black church, where modifications to the TBI were made by church-based health advisors.

### Race and Ethnicity Consciousness

A total of 26.4% (29/110) of the studies met our race or ethnicity consciousness criteria that at least one manuscript section be explicit about a particular URM group. In total, 24% (7/29) of these studies included samples that comprised 100% members of a single URM group and were race or ethnicity conscious across all manuscript sections—acknowledging a racial or ethnic group in the title, introduction, methods, results, and discussion sections (African American or Black individuals [82,120], American Indian or Alaska Native individuals [34-36], and Hispanic or Latino individuals [40,41]). An additional 17% (5/29) of the studies [38,42,44,51,132] were race or ethnicity conscious across all sections except for the manuscript title. Among the remaining 59% (17/29) of the studies, evidence of race or ethnicity consciousness was present yet inconsistent (Table 4).

**Table 4:** Race- and ethnicity-conscious studies by manuscript section.

Study	Title	Introduction	Methods	Results	Discussion
Wechsberg et al [120]	✓	✓	✓	✓	✓
Jordan et al [82]	✓	✓	✓	✓	✓
Gorman et al [35]	✓	✓	✓	✓	✓
Campbell et al [34]	✓	✓	✓	✓	✓
Montag et al [36]	✓	✓	✓	✓	✓
Muroff et al [40]	✓	✓	✓	✓	✓
Muroff et al [41]	✓	✓	✓	✓	✓
Osilla et al [42]		✓	✓	✓	✓
Yang et al [132]		✓	✓	✓	✓
Paris et al [38]		✓	✓	✓	✓
Nayak et al [43]		✓	✓	✓	✓
Nayak et al [44]		✓	✓	✓	✓
Burner et al [51]		✓	✓	✓	✓
Comulada et al [45]	✓	✓	✓		✓
Brusoski and Rosen [121]	✓	✓	✓		
Preston et al [97]		✓		✓	✓
Dawson Rose et al [112]				✓	✓
Forray et al [72]				✓	✓
Saberi et al [114]			✓		✓
Ondersma et al [68]			✓		✓
Reback et al [103]		✓			
Tzilos et al [67]		✓			
Tzilos Wernette et al [54]		✓			
Ondersma et al [70]		✓			
Ondersma et al [75]			✓		
Ondersma et al [77]			✓		
Waller et al [92]			✓		
Suffoletto et al [134]				✓	
Gryczynski et al [140]					✓

### Critical Appraisal

Higher appraisal or confidence logically comes from studies with race- or ethnicity-conscious results with samples that included ≥90% of members of a single URM group (10/110, 9.1%) [34,35,38,40-42,82,120,121,132]. Despite not having race- or ethnicity-conscious results, the samples of 11.8% (13/110) of the studies included ≥90% of members of a single URM group, making the results intrinsically applicable to that group (ie, functionally equivalent to being race conscious) [37,45,63-66,69,104,128,130,131,142]. A small group of studies (8/110, 7.3%) [36,43,44,51,72,97,112,134] had samples comprising <90% of members of a single URM group; however, due to their race- or ethnicity-conscious results, there was higher confidence in interpretable findings. A lower appraisal or confidence rating was assigned to those studies that included <90% of members of a single URM group *and* lacked race-conscious results (79/110, 71.8%). As samples increase in racial or ethnic diversity, without subgroup analyses, it becomes challenging to clearly interpret outcomes for different groups. Table 5 shows the critical appraisal of all the studies in the dataset.

**Table 5:** Critical appraisal of the dataset (N=110)^a^.

Race-conscious results	<50% of participants from a single URM^b^ group	50%-59% of participants from a single URM group	60%-69% of participants from a single URM group	70%-79% of participants from a single URM group	80%-89% of participants from a single URM group	≥90%-100% of participants from a single URM group
Yes	Dawson Rose et al [112]	Suffoletto et al [134]	Nayak et al [43] Forray et al [72] Preston et al [97]	Nayak et al [44]	Montag et al [36] Burner et al [51]	Campbell et al [34]^c^ Gorman et al [35]^c^ Paris et al [38] Muroff et al [40,41] Osilla et al [42]^c^ Jordan et al [82]^c^ Brusoski and Rosen [121]^c^ Yang et al [132]^c^ Wechsberg et al [120]^c^
No	Braciszewski et al [53] Tzilos Wernette et al [54] Carroll et al [55] Sugarman et al [56] Tofighi et al [108] Saberi et al [114] Osilla et al [110] Dennis et al [94] Tofighi et al [107] Shrier et al [116] Shrier et al [117] Delrahim-Howlett et al [106] Hasin et al [83] Dawson Rose et al [113] Ditre et al [139] O’Gurek [127] Castillo et al [125] Kiluk et al [80] Tofighi et al [109] Reback et al [101,102] Reback et al [103] Steers et al [138]	Aharonovich et al [39] Comulada et al [46] Aronson et al [52] Kiluk et al [57] Kiluk et al [58] Muench et al [60] Braciszewski et al [76] Saberi et al [114] Cunningham et al [88] Moore et al [135] Welsh [122] Babson et al [143] Aharonovich et al [86] Kiluk et al [78] Kiluk et al [79] Roos et al [81] Budney et al [105] Gordon et al [129] Walton et al [89] Blow et al [91] Waller et al [92] Drislane et al [93] Kennedy et al [111]	Sanjuan et al [47] Trudeau et al [48] Tolou-Shams et al [49] Thompson et al [59] Alemi et al [62] Hasin et al [84] Glasner et al [136] Moore et al [141] Ingersoll et al [115] Martino et al [71] Loree et al [73] Yonkers et al [74] Aharonovich et al [85] Preston et al [98-100] Walton et al [90] Buu et al [133] Spohr et al [137] Mehtani et al [126]	Mullen et al [50] Aharonovich et al [87] Ondersma et al [75] Scott et al [96]	Ingersoll et al [61] Frueh et al [119] Palfai et al [124] Tzilos et al [67] Ondersma et al [70,77] Gryczynski et al [140] Scott et al [95]	Legha et al [37]^c^ Comulada et al [45] Pollick et al [69]^c^ Ondersma et al [63-65]^c^ Saladin et al [128] Freedman et al [130] Ondersma et al [66] Linas et al [131] Brooks et al [104] Jaconis et al [123]^c^ Beckham et al [142]^c^

^a^Lower appraisal: <90%-100% of participants from a single underrepresented minority group AND NO race-conscious results; higher appraisal: ≥90%-100% of participants from a single underrepresented minority group OR race-conscious results.

^b^URM: underrepresented minority.

^c^100% of participants are from a single underrepresented minority group.

## Discussion

### Health Equity Promotion

#### Overview

To our knowledge, this scoping review is the first systematic literature exploration of health equity promotion in TBIs for substance use treatment among URM groups at heightened risk of disparities in substance use treatment and related outcomes in the United States [6,144-146]. This review offers insights into the extent to which the evaluated studies promote health equity and identifies opportunities for growth. Productive health equity research focuses on strengths and solutions in lieu of redocumenting problems [24,147,148]. All the identified areas of opportunity for growth will include examples for how to promote equity in these spaces.

#### Access or Inclusion

An analysis of US trials in ClinicalTrials.gov [149] revealed that, over the past 2 decades (2000-2020), most registered studies did not report race and ethnicity enrollment data and, among those with demographic data, racial or ethnic minority groups were underrepresented. It is noteworthy that federal requirements to report have only been in place since 2017. In our review, nearly half (532/1158, 45.9%) of the full-text articles assessed for eligibility were excluded due to either ineligibility based on demographic criteria or the absence of reporting of race or ethnicity data, substantiating this historical pattern of lack of access or inclusion that perpetuates health inequity for some of the most vulnerable members of our society. Of those studies that did include URM groups, approximately half (57/110, 51.8%) described engagement of end users in some stage of the TBI research. The scientific community is increasingly focusing on translation and implementation of scientific discoveries with a parallel increase in attention to end-user engagement as a key component in the process of tailoring best practices for specific populations who are hardly reached by research and, thus, disproportionately impacted by chronic health conditions [150,151]. The promotion of representation and equity in research are by-products of these foci [152]. As will be discussed more extensively in the following sections, community-engaged and culturally informed interventions have been demonstrated to improve the health outcomes of underserved groups facing health disparities [26,153,154]. Research collaboration with the communities we seek to serve and partner with to promote health equity through the development of TBIs for substance use is essential.

#### Range and Nature of TBIs

Most of the netted studies (76/110, 69.1%) were programs of research, highlighting the importance of conducting various types of research studies focused on different stages of science or scientific questions (eg, intervention development, efficacy studies, and effectiveness studies), consistent with the National Institutes of Health (NIH) Stage Model for behavioral intervention development, with the ultimate goal of producing highly potent and maximally implementable interventions that improve health and well-being [155]. This model is also consistent with the path to understanding the replicability and reproducibility of effects in various contexts and populations [24]. The independent studies netted by this review are disproportionately more recent publications, pointing to future research programs with commitments to URM group health equity promotion. Although expected given the rapid proliferation in TBIs in health research generally, the increased volume in recent years, as well as the range of substances targeted by the studies in this dataset, are encouraging signs.

The prevalence of independently delivered or accessed TBIs is notable given the potential for many underserved people to live in contexts in which access to care may be limited. In addition, many people who identify as members of URM groups may be reluctant to engage in traditional models of care due to mistrust of the research and medical communities [156-158]. Furthermore, the prevalence of brief evidence-based interventions accessed or delivered independently reduces the threshold to receive an effective dose of the intervention [159]. Efforts to simplify technological complexity are likely to disproportionately benefit less advantaged groups [19,160], possibly due to the effort involved in negotiating additional barriers to health behaviors that emerge in underserved contexts [161].

#### Race and Ethnicity Consciousness

Abandoning color-blind approaches to research potentiates exploration of treatment impacts that values the perspectives and voices of others [162]. Approximately one-quarter of the studies (29/110, 26.4%) were explicitly conscious of members of underrepresented racial and ethnic groups in their research. Most of the race- or ethnicity-conscious studies (23/29, 79%) also engaged end users and community members in the design or conduct of the research, further amplifying their voices. These exemplars stand in contradistinction to acontextually developed innovations that may largely benefit health outcomes in one sector of society while potentially creating, sustaining, or increasing health disparities in another [163].

#### Critical Appraisal

The trend toward an increased focus on including URM groups in TBI research, as well as the increased race and ethnicity consciousness of this research, is unquestionably a positive finding. However, most studies (79/110, 71.8%) were limited in the extent to which the outcomes could be interpreted to have implications for people who identify as African American or Black, Hispanic or Latino, or American Indian or Alaska Native individuals. In our critical appraisal, 28.2% (31/110) of the studies were highly appraised in terms of the interpretability of findings for one or more URM groups. Through this appraisal of the dataset, as well as the scoping review at large, we hope to encourage researchers to identify existing TBIs for potential adaptation, potential gaps in the knowledge base, or insights for new tool development.

### Opportunities for Growth

Careful attention to patterns in the review data illuminate several opportunities for promoting health equity in substance use treatment research with URM groups: lowering access thresholds to increase participation in research, especially among Hispanic or Latino and American Indian or Alaska Native individuals; expanding TBI substance use research focusing on opioids, stimulants, and cannabis; using culturally informed theoretical models or equity approaches to knowledge production; planning and conducting racial and ethnic subgroup analyses; adapting TBIs with demonstrated effectiveness for examination with URM groups; and considering intersectionality.

#### Lowering Access Thresholds to Increase Participation, Especially Among Hispanic or Latino and American Indian or Alaska Native Individuals

To enhance inclusion, low-threshold, community-based recruitment strategies and settings are needed. Most participants in our dataset were recruited from medical centers, substance use treatment settings, or integrated medical and substance use treatment programs in academic settings. These settings are considered high threshold due to access requirements of transportation [164], insurance [165], trust in the medical community [156,157], and time to participate. The increased challenges of conducting research with people who are incarcerated compared with people in the community [166] and the disproportionate burden of incarceration on URM groups [167] suggest reconsideration of barriers to the conduct of research with people who are incarcerated. This is a significantly understudied population and doubly marginalized by URM group and prisoner status (see the Intersectionality section).

Given the prevalence of high-threshold recruitment settings in our dataset, the dearth of research on TBIs for substance use treatment among people who identify as Hispanic or Latino or American Indian or Alaska Native individuals is unsurprising and significant. Both American Indian or Alaska Native and Hispanic or Latino individuals experience some of the worst health disparities in the United States and are more likely than White individuals to be poor and unemployed and have lower educational attainment [145,146,168]. American Indian or Alaska Native individuals are also more likely than White individuals and other URM groups to live in rural frontier settings, compounding the issue of access and inclusion [169]. Recruitment of sufficient numbers of racial and ethnic minority group participants is imperative to understanding how research affects underrepresented groups [25], with underrepresentation limiting the extent to which members of these groups benefit from advances in substance use treatment research [170]. Community-engaged recruitment strategies are needed to increase access and inclusion of URM groups [171].

#### Expanding TBI Substance Use Research Targeting Opioids, Stimulants, and Cannabis

Few netted studies (30/110, 27.3%) targeted opioids, stimulants (eg, methamphetamines and cocaine), and cannabis specifically. The US opioid and stimulant epidemics [172], as well as the increasing legalization of cannabis [173], create an expanding need for treatment for all people navigating problematic or disordered use [174]. However, this is particularly true for URM groups due to their marginalization in *both* treatment and research [175]. TBIs offer the potential to mitigate health-related disparities in care by addressing the needs of members of diverse groups and increasing access. While there is limited work focusing on people who use opioids, stimulants, and cannabis in the United States generally, our review identified the lack of research on TBIs for these drug classes that includes URM groups. The gaps in the literature on TBIs for these 3 drug classes offer enormous opportunities for promoting health equity. Several reviews have summarized the use of TBIs related to substance use [18,176-182]. However, most interventions have focused exclusively on impacting alcohol use behaviors [183].

Expanding TBI development and research beyond a heavy focus on alcohol is especially critical due to the opioid overdose epidemic. Opioid-related overdose deaths among people identifying as African American or Black and American Indian or Alaska Native individuals now outpace those of White individuals in the United States, in part because the science and resources brought to bear to fight the opioid epidemic disproportionately exclude people who identify as belonging to URM groups, especially during the early phase of the epidemic [184,185]. A total of 3.6% (4/110) of the identified studies in this scoping review focused on TBIs for opioid use disorder (OUD); all 4 were telehealth focused [186-189]. While regulations expanding telehealth care for OUD issued during the COVID-19 pandemic appear to remain in place [190], further enabling the exploration of the benefits of telehealth for OUD treatment and developing creative, effective, and culturally tailored TBIs for OUD in addition to telehealth interventions is important to expand access to OUD care.

#### Using Culturally Informed Theoretical Models or Equity Approaches to Knowledge Production

The theoretical models and perspectives represented in the dataset were predominantly generalizability approaches, with negligible use of culturally informed or sensitive theories or equity approaches to knowledge production (eg, sociological trust theory, community-based participatory research, public health critical race praxis, and social determinants of health) [19,163,191,192]. While the identification of the commonalities of human experience is useful, to promote health equity, researchers must explore the differential effectiveness of evidence-based interventions for URM groups.

Several netted studies (4/110, 3.6%) [34,40,41,82] used equity-promoting theories or frameworks based on community-engaged, participatory research practices in adaptations [40,41,82] or explorations of the feasibility of the adaptation [34] of evidence-based TBIs. An understanding of the power of culturally or contextually informed theories and frameworks to disrupt social mechanisms that fuel health disparities may be instructive. For example, the theoretical underpinnings for the Addiction Comprehensive Health Enhancement Support System [40,41] are based on self-determination theory [193]. One of the defining features of self-determination theory is its treatment of both the person and the social context in motivated behavior. By adapting and translating the Addiction Comprehensive Health Enhancement Support System to be linguistically and culturally relevant to Spanish-speaking Latinos in recovery, the authors support competence and autonomy by eliciting and acknowledging patients’ context and perspectives [194].

Community-based participatory research methods incorporate community member input at all phases of the research process [163]. Offering an evidence-based treatment (CBT4CBT) in an alternative setting, such as a Black church, Jordan et al [82] explored the role of context to improve access to and the efficacy of evidence-based treatments. Through engagement with the community, as well as training church staff to deliver evidence-based treatments, the authors assessed feasibility and probed necessary adaptations to the TBI to improve acceptability. Campbell et al [34] used community-engaged research methods to reveal preferences regarding delivery and presentation, including using American Indian or Alaska Native actors for voice and video components, use of stories as opposed to “academic” presentations, and inclusion of American Indian or Alaska Native cultural representation. There is substantial evidence-based work in the field of TBIs for substance use treatment. Using theories and frameworks that are sensitive to context and the diversity of human experiences, community-engaged research methods, and adaptations is possible, and efficacy is likely to be strengthened for members of underrepresented groups.

#### Planning to Conduct Racial and Ethnic Subgroup Analyses

Limited subpopulation analyses pose significant problems for reviews [159]. Of the 110 studies in this review, 81 (73.6%) missed an opportunity to better understand the race or ethnicity subgroup–specific implications of their findings. Thus, while it may appear that the studies identified in our review focused on TBIs with a strong evidence base, more nuanced assessments of how differentially effective these standard-bearers might be is lacking. Given the limited inclusion of members of underrepresented populations in biomedical research [195], when a sample does include sufficient representation, it is a prime opportunity for interested researchers to review data to improve our understanding of the implications for these groups. Burlew et al [25] further recommend that researchers consider within-race differences and the potential disadvantages of combining racial or ethnic minority groups in the analyses (eg, Weaver et al [196] revealed differential odds of meeting the criteria for major depressive disorder between rural Black women [lower] and urban Black women [higher]).

There is opportunity to explore clinical trials with diverse samples or large datasets that are a part of the public record to help identify moderators to improve our understanding of the differential impacts of our policies and interventions for URM groups and the multiply disadvantaged among us [197]. The NIH recently (2021) recommended that investigators should not control for the contextual variables (eg, neighborhoods and employment opportunities) that are the drivers of inequity but, rather, that these factors be explicitly studied. Systems of oppression are inherently bound together—the intersection of multiple identities (eg, gender and race) within social systems of power may compound and synergistically exacerbate experiences of health disparity [198].

#### Adapting TBIs With Demonstrated Effectiveness for Examination With URM Groups

Related to the aforementioned opportunity for growth regarding planned subgroup analyses, another opportunity for interested researchers is to adapt TBIs with demonstrated effectiveness for examination among URM groups. Many of the programs of research netted in this review underscore this opportunity. For example, Paris et al [38] described the primary outcomes of a randomized clinical trial comparing a culturally adapted version of CBT4CBT-Spanish to standard outpatient mental health and addiction treatment in a heterogeneous population of treatment-seeking Latino adults. Their rationale for undertaking this research included consideration of previous findings on the efficacy, safety, and durability of the existing English-language versions of CBT4CBT when added to standard outpatient treatment. There is no shortage of TBIs for substance use disorders; however, as this review reveals, there is a shortage of understanding which among the many might be efficacious for URM groups.

#### Considering Intersectionality

While seeking to characterize the range and nature of TBIs for substance use treatment among people who identify as African American or Black, Hispanic or Latino, and American Indian or Alaska Native individuals, we discovered that most of the studies (85/110, 77.3%) included historically underserved populations, such as pregnant or perinatal women, people living with HIV or AIDS, adolescents or youth, youth exiting foster care, people who are justice or court involved, people who are unhoused, and veterans. In addition, most of the participants across these studies were impacted by multiple socioeconomic challenges, notably income, housing, education, and health insurance. A recent literature review on multiple disadvantages in relation to telehealth [199] identified intersectionality theory [200] as an approach that the authors felt might help account for digital disparities. Intersectionality theory posits that considering a single axis of inequality is limited and that considering disadvantage on multiple axes simultaneously is needed [201]. Applied to digital health disparities, this theory suggests the hypothesis that our individual identities and lived experiences are unique and multidimensional and that we will or will not use TBIs based on our unique identities and experiences rather than due to our identification with single categories such as race, age, gender, or disability. Per intersectionality theory, addressing health inequities will entail an increased understanding of how various structural and social determinants of health interact. To achieve this increased understanding, we must work with individuals, families, groups, and communities to understand what shapes their experiences of advantage and disadvantage. The import of this theory cannot be understated for the data that we collected. To capture intersectionality, a first challenge for researchers working in this space and beyond is to take a step back, digest the large-scale studies that appear to have uniform messages about broad factors associated with efficacy or effectiveness applicable to all [202], and commit to collecting and reporting data related to multiple categories at once (eg, race, sex, and income). A second challenge is to combine this knowledge with tailored implementation strategies, acknowledging how different aspects of disadvantage related to social determinants of health interact in individual lives, impacting access, adoption, and effectiveness of TBIs for substance use. Dr Nora Volkow, the director of the National Institute on Drug Abuse, recently blogged that “measuring social determinants of health can help researchers better design treatment interventions and services, as well as make addiction care more equitable. Research in other areas of medicine has already revealed the distorting effects of failure to take that step” [203]. We must strive to conduct research that promotes health equity for all.

### Future Research

We acknowledge researchers who have been working in the health equity field that dates back more than a century [148]. While most of our team members identify as White, we acknowledge the historical damage posed by many White allies and aspire beyond “health equity tourism” [148,204]. We aim to be solution focused, collaborating with deeply knowledgeable equity researchers to build on the knowledge presented herein. In this spirit, we seek to partner with scholars from underrepresented groups on future research exploring optimal practices for research with diverse and underrepresented groups; race and ethnicity consciousness; and effectiveness of TBIs for people who identify as African American or Black, Hispanic or Latino, and American Indian or Alaska Native individuals based on this scoping review. We encourage readers to take note of the research opportunities implied in the Opportunities for Growth section as well.

### Limitations

Several limitations warrant attention. First, our eligibility criteria of samples of ≥50% of URM group members may have precluded exploration of literature with the potential to provide further insights to promote health equity in the TBI for substance use treatment arena. Second, the threshold for designation of higher appraisal or confidence versus lower appraisal confidence of 90% of members of a single URM group in the study samples was an arbitrary cutoff and, as such, highlights the guidance nature of critical appraisals. Per a recent publication on critical appraisals, “decisions on whether a study is considered weak, moderate, or strong are based on arbitrary cut-off scores” [205]. In theory, any cutoff of >50% would show that most participants were from a single URM group, although 90% could be considered a conservative cutoff to ensure that the results have direct implications for that group. The appraisal is intended as guidance or a gauge of the confidence that a reader should reasonably have that the study in question may have interpretable findings for a particular URM group. Third, to avoid intervention-generated inequalities and ensure that an equity lens is applied in the conduct, reporting, and use of research, an oft-cited recommendation is for researchers to report relevant sociodemographic characteristics of the samples. These include the PROGRESS-Plus (place of residence, race, ethnicity, language, occupation, gender or sex, religion, educational level, SES, social capital) factors of place of residence, race, ethnicity, language, occupation, gender or sex, religion, educational level, SES, age, disability, and sexual orientation [19,206]. This framework functions as a reminder that understanding health equity encompasses numerous factors beyond race and ethnicity that impact intervention access, adherence, adoption, and effectiveness. While our review focused on race or ethnicity, we acknowledge the importance of attending to a broader array of demographic variables to ensure that TBIs do not fall into the trap of inadvertently contributing to increasing health inequities, increasing the gap between the most and least advantaged. Fourth, a general limitation of the TBI research included in this review was the low SES among participants of the netted samples, as well as the high-threshold venues for recruitment. Both are filters that limit a more thorough understanding of the potential of TBIs to promote health equity.

### Conclusions

Through this review, we sought to provide context, insights, and direction for researchers working to develop and evaluate digital technology substance use interventions while promoting health equity. An NIH recommendation on enhancing health disparity research suggests that we must go beyond “ticking the box” when including racial and ethnic minority groups in research [158]. Instead, underrepresented populations must be *the focus* of studies, or at the very least, the studies must be race and ethnicity sensitive [197]. As we do not all experience equity in health, we must resist the temptation to use universal instruments to attain universal ends; one size has never fit all [207]. This scoping review was conducted in the spirit of this recommendation. We sought to focus attention on those studies that included members of underrepresented groups and, further, those that gave voice to these people through race- and ethnicity-conscious research or end-user engagement. A noted sociologist who writes about technology and its potential to exacerbate inequities suggests that “the ways we engineer the material world reflects and reinforces (but could also be used to subvert) social hierarchies” [208]. It is our fervent hope that insights into the engineering of TBIs for substance use treatment for URM groups can and will be used to erode structural racism in health care.

## Appendix

Multimedia Appendix 1 PRISMA (Preferred Reporting Items for Systematic Reviews and Meta-Analyses) checklist.

Multimedia Appendix 2 MEDLINE electronic search strategy.

## Abbreviations

CBT: cognitive behavioral therapy
CBT4CBT: Computer-Based Training for Cognitive Behavioral Therapy
CM: contingency management
EMA: ecological momentary assessment
MES: Motivation Enhancement System
NIH: National Institutes of Health
OUD: opioid use disorder
PRISMA-ScR: Preferred Reporting Items for Systematic Reviews and Meta-Analyses extension for Scoping Reviews
SES: socioeconomic status
TAU: treatment as usual
TBI: technology-based intervention
URM: underrepresented minority
